# Clofazimine-Mediated, Age-Related Changes in Skeletal Muscle Mitochondrial Metabolites

**DOI:** 10.3390/metabo13050671

**Published:** 2023-05-19

**Authors:** Jennifer Diaz-Espinosa, Kathleen A. Stringer, Gus R. Rosania

**Affiliations:** 1 Department of Pharmaceutical Sciences, College of Pharmacy, University of Michigan, 428 Church Street, Ann Arbor, MI 48109, USA; jendiaz@umich.edu (J.D.-E.); grosania@med.umich.edu (G.R.R.); 2 Department of Clinical Pharmacy, College of Pharmacy, University of Michigan, 428 Church Street, Ann Arbor, MI 48109, USA; 3 Division of Pulmonary and Critical Care Medicine, Department of Medicine, School of Medicine, University of Michigan, Ann Arbor, MI 48109, USA; 4 Weil Institute for Critical Care Research and Innovation, University of Michigan, Ann Arbor, MI 48109, USA

**Keywords:** adverse drug reactions, mitochondrial metabolism, l-carnitine, acetylcarnitine, cardiac muscle

## Abstract

Mitochondrial health declines with age, and older patients can demonstrate dysfunction in mitochondrial-rich tissues, such as cardiac and skeletal muscle. Aged mitochondria may make older adults more susceptible to adverse drug reactions (ADRs). We assessed mitochondrial metabolic function by measuring two metabolites, l-carnitine and acetylcarnitine, to determine their effectiveness as candidate clinical biomarkers for age-related, drug-induced alterations in mitochondrial metabolism. To study age- and medication-related changes in mitochondrial metabolism, we administered the FDA-approved mitochondriotropic drug, clofazimine (CFZ), or vehicle for 8 weeks to young (4-week-old) and old (61-week-old) male C57BL/6J mice. At the end of treatment, whole blood and cardiac and skeletal muscle were analyzed for l-carnitine, acetylcarnitine, and CFZ levels; muscle function was measured via a treadmill test. No differences were found in blood or cardiac carnitine levels of CFZ-treated mice, but CFZ-treated mice displayed lost body mass and alterations in endurance and levels of skeletal muscle mitochondrial metabolites. These findings demonstrate the age-related susceptibility of the skeletal muscle to mitochondria drug toxicity. Since drug-induced alterations in mitochondrial metabolism in skeletal muscle were not reflected in the blood by l-carnitine or acetylcarnitine levels, drug-induced catabolism and changes in muscle function appear more relevant to stratifying individuals at increased risk for ADRs.

## 1. Introduction

Mitochondrial dysfunction is a hallmark of aging, which leads to changes in metabolism and manifests as structural and functional impairments in various organ systems, including muscle, which has a high energy demand [1,2,3]. Many medications that accumulate in mitochondria can also cause disruptions in mitochondrial function [4,5]. The combination of age-related declines in mitochondrial function could enhance the susceptibility to adverse drug reactions (ADRs) to mitochondriotropic drugs, particularly those with known effects on cardiac and skeletal muscle function [6,7]. In these cases, elderly patients may be at higher risk of drug toxicity affecting muscle function resulting from mitochondrial drugs compared to the younger population [8]. Hence, there is a need to identify biomarkers for monitoring drug mitochondriotoxicity in elderly patients.

Many FDA-approved medications are cell membrane-permeant small molecules that concentrate in organelles, such as lysosomes and mitochondria, via physicochemical mechanisms involving pH and ion gradients across the membranes bounding these organelles [9,10]. Lysosomes and mitochondria are both important organelles for lipid metabolism and energy production [11]. Mitochondria are involved in lipid biosynthesis and synthesize large amounts of ATP through fatty acid beta-oxidation that is dependent on the carnitine shuttle, where l-carnitine gets converted to acetylcarnitine [12]. Lysosomes are important organelles for phospholipid uptake into cells via the endocytosis of low-density lipoprotein particles and lipid degradation by lysosomal lipases [13].

To study age-related differences in medication-induced alterations in mitochondrial-related metabolism, we used the mitochondriotropic and lysosomotropic drug clofazimine (CFZ), an FDA-approved antibiotic that accumulates in both lysosomes and mitochondria, with effects on both lysosomal and mitochondrial structure and function [14,15]. As an easily accessible systemic biomarker of mitochondrial and lysosomal changes induced by drugs that accumulate in these organelles, l-carnitine and its acetylated metabolite, acetylcarnitine, have been proposed as clinically relevant biomarkers to detect patients at increased risk of serious ADRs, especially among the elderly [5,16]. While there are numerous mitochondrial metabolites, these attributes make l-carnitine and acetylcarnitine viable candidate biomarkers for ADRs [5]. Therefore, to further understand the relationship between age and medication exposure and mitochondrial metabolic function, we used measurements of l-carnitine and acetylcarnitine, and accompanying changes in total body mass, food intake, muscle mass and endurance.

Metabolite measurements were taken of the blood, skeletal and cardiac muscles of young and old C57BL/6J mice treated with either CFZ or vehicle.

## 2. Materials and Methods

### 2.1. Animal Studies

Animal use protocols were approved by the University of Michigan’s Institutional Committee (PRO00007593; 5 May 2017, PRO00009404; 13 December 2022), and animal care was provided by the University of Michigan’s Unit for Laboratory Animal Medicine (ULAM), where animals were maintained at 22 ± 2 °C and a relative humidity of 55 ± 5%, in a standard 12 h dark–light cycle, and had unconstrained access to water and food. In accordance with ULAM and NIH guidelines, young (4 weeks) and old (61 weeks) male C57BL/6J mice from Jackson Laboratory (Bar Harbor, ME, USA), did not require acclimation prior to experimentation. These mice were randomized to receive either CFZ (C8895, Sigma-Aldrich, St. Louis, MO, USA) to achieve a dose of 36 mg/kg/day or equal amounts of the vehicle sesame oil (720189601668, Kadoya, Tokyo, Japan) for 8 weeks. At the end of treatment, food consumption was monitored via specialized cages (3700M022, Tecniplas, Buguggiate, Italy) for 18 h, after which animals were euthanized by CO_2_ inhalation following a cardiac puncture for blood sample acquisition in compliance with American Veterinary Medical Association Guidelines. Immediately after euthanasia, the heart and gastrocnemius (GAS) muscles were isolated, washed with 1X phosphate-buffered saline (PBS), blotted dry, and flash-frozen in liquid nitrogen. While experiments were conducted in compliance with the Animal Research: Reporting of In Vivo Experiments guidelines [17], it should be noted that true blinding of the experiments was not feasible due to the red pigmentation in the skin induced by CFZ. This was readily apparent at the time of experiment termination. In addition, CFZ induces readily apparent changes at the molecular level of the organ tissue (e.g., CFZ crystals) that do not permit sustained blinding.

### 2.2. Quantification of Carnitine, Acetylcarnitine and CFZ

Quantification of l-carnitine, acetylcarnitine, and CFZ in whole blood, GAS, and heart, with randomization of the samples prior to assay, was performed at the Pharmacokinetic and Mass Spectrometry Core (University of Michigan). Detailed information for each assay is given in the Supplementary Materials. In brief, the muscle was homogenized in n-dimethylformamide-PBS solution. Acetonitrile was added to the whole blood and muscle homogenate for protein precipitation and CFZ extraction. For both assays, concentrations (μg/mL) were derived from an internal standard as measured by liquid chromatography (LC)-mass spectroscopy (MS)/MS. Resulting data were processed using Analyst (1.6, SCIEX, Framingham, MA, USA) for CFZ and SCIEX OS (2.1.6, SCIEX, Framingham, MA, USA) for l-carnitine and acetylcarnitine and a blinded analysis of acetylcarnitine and carnitine data was conducted. To convert CFZ muscle data from μg/mL to μg, concentration values were multiplied by the mass (g) of each sample. Blood concentration data were converted from µg/mL to µM by dividing concentration (µg/mL) by molecular weight (l-carnitine, 161.2; acetylcarnitine, 203.2; clofazimine, 473.4) and multiplying by 1000.

### 2.3. Endurance Testing

Treadmill testing is a reproducible, non-invasive method to assess skeletal muscle function and endurance [18,19]. Mice were randomized for testing, and at the time of testing, each mouse was placed in its own lane on a treadmill (1050-RM, Columbus Instruments). Up to four mice were tested simultaneously and each one ran to exhaustion [20]. Each mouse had a two-minute warm-up session before the endurance treadmill study. The mice ran eight meters per minute with no pulse shocks during the first minute of the warm-up session. For the remaining minute of the warm-up session, pulse shocks were initiated (200-millisecond pulses with a repetition rate of 4 pulses per second and a shock intensity at 3.4 milliamps). The endurance test started immediately after the warm-up session (with the pulse shocks on) and the treadmill speed was set to eight meters per minute. The treadmill speed was increased by one meter every minute. The mice were deemed exhausted if they were on the shock grids for greater than three consecutive seconds without attempting to reengage the treadmill, spent greater than 50% of their time on the shock grid, or at the third time the animal was willing to withstand 2 s or more on the shock grid rather than returning to the treadmill.

### 2.4. Chemical Imaging of CFZ Accumulation in Muscles

Confocal Raman microscopy analysis, as previously described [21], was used to assess the protonation state (freebase or hydrochloride salt) of CFZ that accumulated in the heart and GAS from cryosections (10 μm thick on glass slides). Briefly, a Raman microscope (alpha300R, WiTec) coupled to a 532 nm laser and a Zeiss EC EPIPLAN 50x objective (N.A. = 0.75) was used to acquire large-area scans (50 μm by 50 μm) with a step size of 50 μm and an integration time of 0.1 ms per pixel throughout the sections. The WiTec Project FOUR software was utilized to remove cosmic rays, and a MATLAB^®^ processing algorithm developed in-house was used to baseline-subtract, normalize, and generate the differences between spectra. Muscle spectral references were gathered from CFZ-crystal negative regions and true muscle reference spectra were gathered from vehicle-treated sections. Sample spectra were compared to the reference spectra of pure CFZ-freebase (CFZ-FB) and CFZ-hydrochloride (CFZ-HCL) to assess the protonation state of the drug present in association with the tissue samples.

### 2.5. Statistics

Data were compared using either a one-way ANOVA followed by post-hoc Tukey’s test when the ANOVA *p* value was significant, or an unpaired Student’s *t*-test with equal variance, when comparisons between two groups were made; no data were excluded. Statistical analyses and associated graphics were constructed using PRISM (9.4.1; GraphPad Software, Inc., San Diego, CA, USA), where a *p*-value of ≤0.05 was considered significant. Based on prior work using a standard deviation range of 10–15 [22], an α of 0.05, and a power of 80%, we calculated a sample size of between 10 and 17 mice per group.

## 3. Results

### 3.1. Age and CFZ-Related Changes in Muscle Mitochondrial Metabolites Are Not Reflected in the Blood

**Figure 1:**
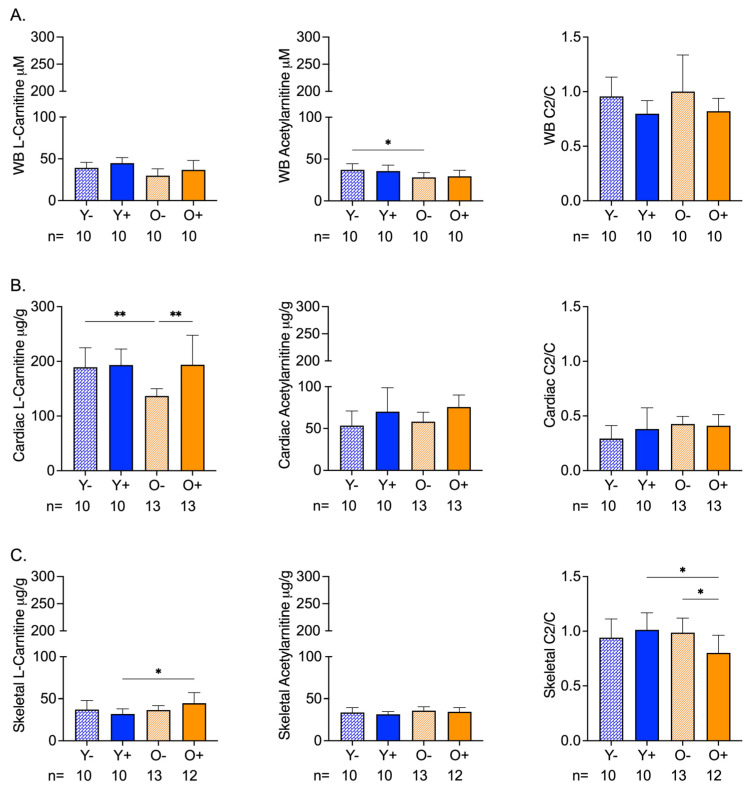
Carnitine (C) and Acetylcarnitine (C2) Levels in Whole Blood and Cardiac and Skeletal Muscles of Aged and CFZ-Treated Mice. (**A**) Whole blood C and C2 levels and C2/C ratios (mean ± SD). Old vehicle-treated (O−) mice had lower C2 levels than young vehicle-treated (Y−) mice (* *p* = 0.03, Tukey). The C2/C ratios of aged and CFZ-treated were not different across groups. (**B**) Cardiac muscle C and C2 levels and C2/C ratios (mean ± SD). C decreased in O− mice compared to Y− (** *p* = 0.01, Tukey), where C was higher in old CFZ-treated mice (O+) than in the negative control, O− (** *p* = 0.001, Tukey). CFZ treatment and age did not change the C2/C ratios. (**C**) Skeletal muscle C and C2 levels and C2/C ratios (mean ± SD). CFZ treatment increased C levels in old mice (O+) compared with young mice, Y+ (* *p* = 0.01, Tukey), but neither were different from their respective negative control. CFZ treatment decreased the C2/C ratio in old (O+) mice compared to young (Y+) mice (* *p* = 0.01, Tukey) and vehicle-treated old O- mice (* *p* = 0.02, Tukey). The number of mice per group is shown below the *x*-axis of each plot.

The chemical analysis of whole blood samples revealed age-related differences in acetylcarnitine levels that were not altered by CFZ treatment (Figure 1A). Neither age nor CFZ treatment altered the whole-blood l-carnitine or acetylcarnitine/carnitine ratios (Figure 1A).

Cardiac levels of l-carnitine in old vehicle-treated (O−) mice were lower than the levels in young vehicle-treated (Y−) mice; however, the acetylcarnitine levels remained unchanged (Figure 1B). CFZ treatment resulted in higher l-carnitine and acetylcarnitine levels in old mice (O+) compared with the negative control (O−) (Figure 1B). The acetylcarnitine to l-carnitine ratios of the cardiac muscle did not differ by age or CFZ treatment (Figure 1B).

Skeletal muscle carnitine levels were not affected by age (Figure 1C). However, CFZ treatment resulted in a greater change in skeletal muscle l-carnitine in old (O+) mice than in young (Y+) mice (Figure 1C). There was no change in acetylcarnitine among the groups (Figure 1C). Old CFZ-treated mice (O+) had a lower acetylcarnitine to l-carnitine ratio compared to young CFZ-treated mice (Y+) and old vehicle control mice (O−), demonstrating a more profound CFZ-induced alteration in mitochondrial-related metabolism in old mice (Figure 1C).

### 3.2. Both Young and Old CFZ-Treated Mice Exhibited a Pronounced Catabolic Phenotype Compared with Control Vehicle-Treated Mice

**Figure 2:**
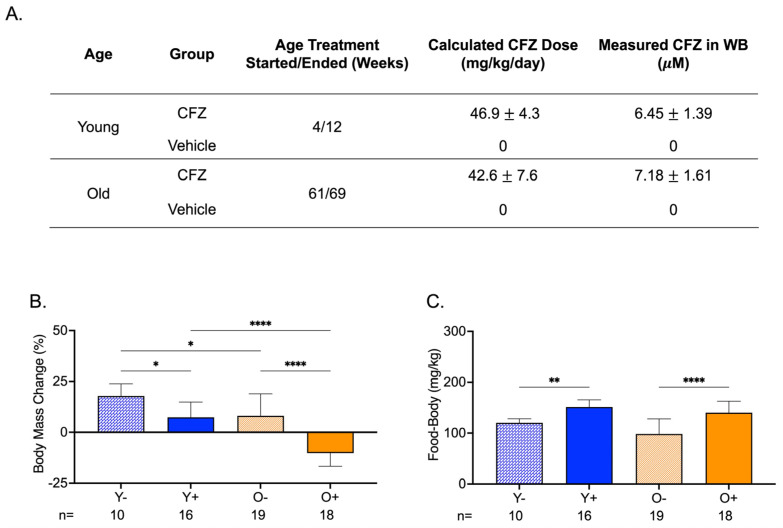
Treatment Groups, Body Mass Changes, and Food Consumption During CFZ Treatment. (**A**) Treatment groups, CFZ dose (mean ± SD), and CFZ in whole blood (mean ± SD). CFZ treatment did not result in different drug doses (*p* = 0.25, Student’s *t*-test) or drug concentrations in whole blood (*p* = 0.10, Student’s *t*-test) in young (Y+) and old (O+) mice. Drug dose was calculated using the terminal body mass and amount of drug-laced food consumed during an 18 h food consumption study conducted prior to euthanasia. Whole blood (WB) CFZ concentration was determined by LC-MS. (**B**) Body mass change (mean ± SD) from the start to the end of treatment. Old mice (O−) gained less weight than young mice (Y−) (* *p* = 0.02, Tukey). Of the CFZ-treated mice, old mice (O+) showed a greater change in percent body mass than young (Y+) mice (**** *p* < 0.0001, Tukey). Young, CFZ-treated mice (Y+) had a smaller mass change than their negative control, Y− (* *p* = 0.01, Tukey), and old CFZ treated mice (O+) showed a decline in percent body mass change compared to the negative control, O− (**** *p* < 0.0001, Tukey). (**C**) Food-to-body ratio (mean ± SD), food consumption (mg) corrected for body weight (kg), was calculated using food consumption measurements made before euthanasia and the terminal body mass. Both young (Y+) and old (O+) CFZ-treated mice consumed more food than their respective controls, Y− (** *p* = 0.005, Tukey) and O− (**** *p* < 0.0001, Tukey). The number of mice per group is shown below the *x*-axis of each plot.

CFZ treatment was initiated in mice at 4 weeks of age (young) or at 61 weeks of age (old). Both groups of mice received the same average CFZ dose in their feed for 8 weeks. The mean whole blood CFZ concentration was not different between the two groups (Figure 2A). The body mass change from the start to the end of treatment was larger in young control mice (Y−) than in young CFZ-treated mice (Y+) and old control mice (O−) (Figure 2B). Old CFZ-treated mice (O+) lost body mass, and the absolute change was greater than in young CFZ-treated (Y+) and old vehicle-treated (O−) mice (Figure 2B). Even though the CFZ-treated groups (Y+ and O+) had smaller body masses at the end of treatment compared to their controls (Y− and O−), the CFZ-treated mice consumed more food than their vehicle controls (Figure 2B,C).

### 3.3. CFZ Treatment Decreased Endurance in Old Mice

**Figure 3:**
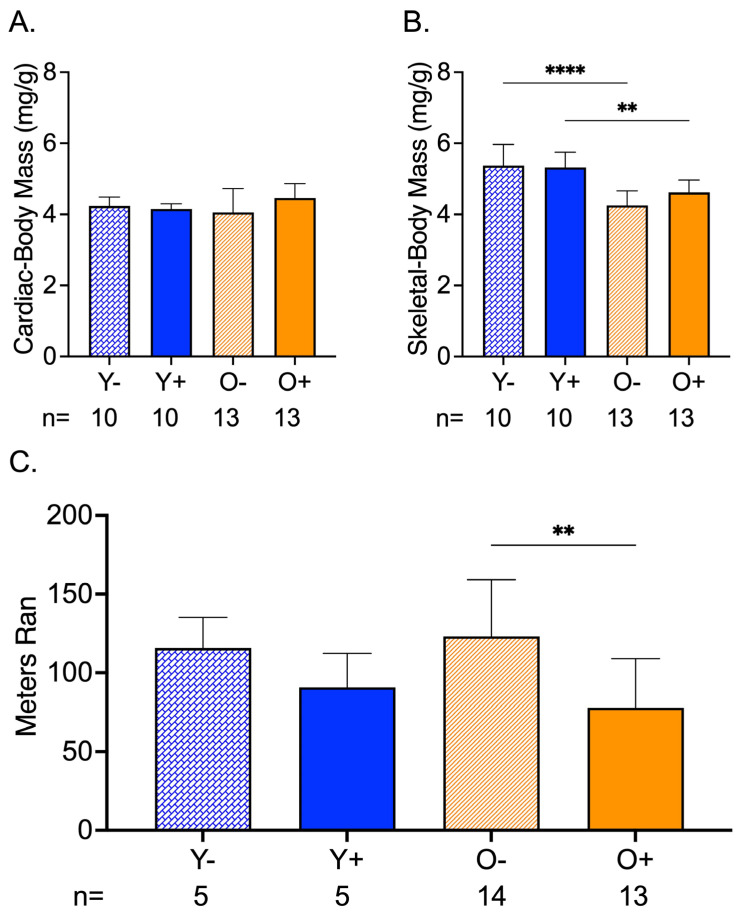
CFZ and Age-Induced Changes in Cardiac and Skeletal Muscle Mass and Endurance. (**A**) Cardiac mass (mean ± SD) corrected for body mass was not different across treatment groups. (**B**) When accounting for body mass, old vehicle-treated (O−) mice had lower skeletal muscle mass compared to young, vehicle-treated Y− mice (**** *p* < 0.0001, Tukey). This finding was sustained in mice treated with CFZ (** *p* < 0.003, Tukey). (**C**) Endurance (mean ± SD), as measured by distance ran in a forced treadmill running study, was reduced by CFZ treatment in old (O+) mice compared with their negative control, O− (** *p* = 0.003; Tukey). The number of mice per group is shown below the *x*-axis of each plot.

Cardiac mass corrected by body mass was not different among the groups (Figure 3A). The normalized skeletal mass by body mass showed an age-related change between Y− and O−, and Y+ and O+, where old mice had lower skeletal-to-body ratios than the young mice (Figure 3B). In old CFZ-treated mice (O+), this translated to a reduction in endurance compared to the negative control, O− (Figure 3C).

### 3.4. Greater CFZ Accumulation Occurred in Skeletal and Cardiac Muscle of Old Mice

**Figure 4:**
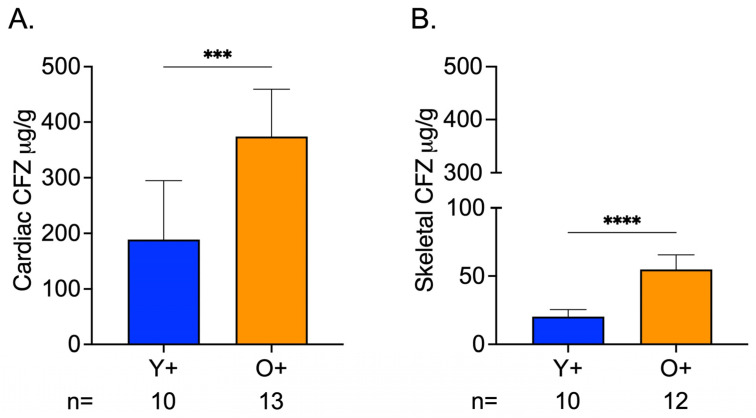
CFZ Accumulation is Greater in the Skeletal and Cardiac Muscle of Old Versus Young Mice. (**A**) CFZ concentration in cardiac muscle per gram of tissue. (**B**) CFZ concentration in skeletal muscle per gram of tissue. Based on LC-MS analysis, CFZ concentration (mean ± SD) in the cardiac (n = 10–13/group) and skeletal muscle (n = 10–12/group) revealed that old mice had a significantly higher CFZ concentration in the cardiac (*** *p* = 0.0001, Student’s *t*-test) and skeletal (**** *p* < 0.0001, Student’s *t*-test) muscles. The number of mice per group is shown below the *x*-axis of each plot.

CFZ accumulation in cardiac and skeletal muscle was influenced by age, where CFZ per gram of tissue was 2.0 times higher in the cardiac muscle and 2.7 times higher in the skeletal muscle of old mice compared to young mice (Figure 4A,B). Not surprisingly, this translated to a higher total amount of CFZ in the cardiac (Y+ = 18.5 ± 10.7, O+ = 50.8 ± 10.1; **** *p* < 0.0001, Tukey) and skeletal (Y+ = 2.5 ± 0.6, O+ = 7.8 ± 1.8; **** *p* < 0.0001, Tukey) muscle of old mice compared to young mice. Given this finding and since CFZ is known to accumulate in the macrophages, we were prompted to assess macrophage numbers in residual skeletal muscle specimens (see Supplementary Materials). We found that there was no difference in macrophage infiltration between CFZ-treated young and old mice (Figure S1A–C). The CFZ concentrations quantified in the muscles of the control mice, Y- and O-, were negligible.

### 3.5. Chemical Analysis of Skeletal and Cardiac Muscle Reveals CFZ Is Present in Discrete, Microscopic Crystalline Inclusions as the Protonated Salt Form of the Drug 

**Figure 5:**
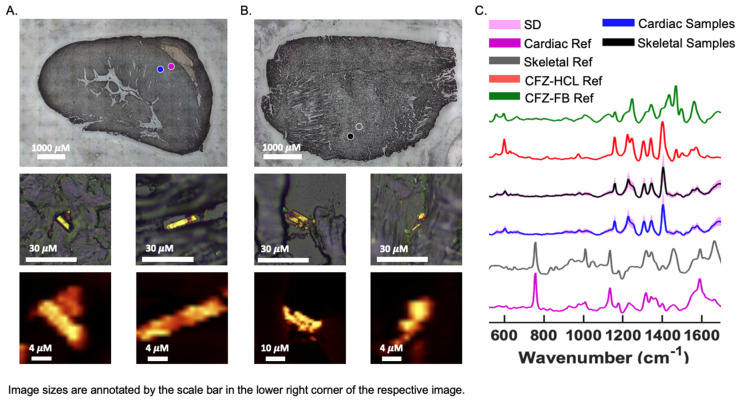
CFZ Occurs Predominantly in the Protonated Form in Skeletal and Cardiac Muscle. Raman bright field (BF) images of a cryo-section of (**A**) cardiac and (**B**) skeletal muscle from a CFZ-treated mouse that show positive CFZ-crystal area (blue dot) in relation to a negative CFZ-crystal area (purple dot). Negative CFZ-crystal areas were scanned to generate (**C**) cardiac muscle reference (purple) (n = 5) and skeletal reference spectra (gray) (n = 5). Raman heat maps (1400 cm^−1^) of CFZ-positive areas in the cardiac and skeletal muscle are shown under their corresponding positive CFZ-crystal BF image. (**C**) CFZ-treated mice spectral signals (mean ± SD, SD = pink outline) from CFZ-positive areas of cardiac (blue spectrum) and skeletal (black spectrum) muscles reveal no indication of the neutral form of CFZ (CFZ-FB, green spectrum), but rather it is present entirely as the protonated salt form (CFZ-HCL, red spectrum) (n = 5/group). This protonated salt form was exclusively detected in both muscle types.

Brightfield (BF) images of CFZ-treated cardiac and skeletal muscles revealed crystal-like structures, smaller than 20 μM, within the sections, which can fit inside cells (Figure 5A,B) [14]. The crystals resembled the protonated form of CFZ, CFZ-hydrochloride (CFZ-HCL). By analyzing muscle tissue sections with a Raman confocal microscope, the Raman-generated heat maps, using the 1400 cm^−1^ signature peak of CFZ-HCL, revealed CFZ was present as the protonated salt form, within microcrystalline structures in the muscle tissue (Figure 5A,B); this is consistent with our previous findings [21]. Based on the size and Raman spectra of the CFZ in the cardiac and skeletal muscles, the spectral evidence indicates that the drug was in the protonated, charged form, which is associated with intracellular (mitochondrial and lysosomal) drug distribution. There were no peaks associated with the presence of the neutral, freebase form of the drug (CFZ-FB), which is the form that typically partitions into body fat and adipose tissue (Figure 5C) [15,23]. To determine whether the muscle reference spectra from crystal-free regions contained CFZ-FB, the reference spectra of muscles from the vehicle control mice were subtracted (Figure S2A,B), demonstrating that the freebase signal was absent from CFZ-treated muscle spectra. 

## 4. Discussion

To understand the medication-induced alterations in the mitochondrial-related metabolism, we used a mitochondriotropic drug, CFZ, in young and old mice to assess the utility of the mitochondrial metabolites, l-carnitine and acetylcarnitine, as biomarkers of drug-induced changes in mitochondrial function [24]; the choice of these metabolites was influenced by our prior work that identified a CFZ-induced alteration in blood levels of l-carnitine [22]. Since the musculature is highly affected by age-related energy metabolism decline [5,25,26], we studied cardiac and skeletal muscle. We chose the gastrocnemius as a representative skeletal muscle because it has been shown to demonstrate the same age-related changes in structure and metabolism as other skeletal muscle types [27], while also being important for mobility and endurance [28]. We measured the function of the gastrocnemius and heart simultaneously via a treadmill test in both untreated and CFZ-treated mice.

Unlike the cardiac muscle, skeletal muscle sustained a drug-induced disruption in l-carnitine homeostasis, as measured by the acetylcarnitine/l-carnitine ratio [29], and a loss of body mass-adjusted mass. This occurred despite a lower per-gram tissue accumulation of CFZ compared with the cardiac muscle. Notably, the lack of a detectable difference in blood levels of l-carnitine or acetylcarnitine in CFZ-treated mice was unexpected, given that muscle is a primary site of mitochondrial metabolism, and there were drug-induced changes in endurance and total body mass. Collectively, these findings suggest that in old individuals, skeletal muscle may be more vulnerable to mitochondria drug toxicity, a problem that does not appear to be readily identified by measurements of blood levels of highly abundant mitochondrial-related metabolites, but is better reflected by a decrease in total body mass in the presence of an increase in caloric intake (catabolism).

Age-related changes in muscle mass give insights into the metabolic vulnerability of skeletal muscle and the metabolic flexibility of cardiac muscle. We have previously shown that 8 weeks of CFZ treatment induces a catabolic state [22]. This was evident in our current study and was more pronounced in old mice (Figure 2B). Catabolism is known to target fat and skeletal muscle mass [30], and spare vital organs like the heart. Specifically, the heart can use a range of substrates to maintain adenosine triphosphate (ATP) production [31], whereas skeletal muscle serves as an important source of amino acids during times of metabolic stress [32]. Our findings suggest that skeletal muscle is more vulnerable to CFZ-induced catabolism in old mice, so much so that it resulted in an organ-level disruption in l-carnitine homeostasis (Figure 1C), loss of mass (Figure 3B), and a reduction in physical endurance (Figure 3C). Again, this did not translate to a detectable change in blood levels of either l-carnitine or acetylcarnitine (Figure 1A).

The bioaccumulation of CFZ in both cardiac and skeletal muscle also provides some clues about our reported age-related differences. Old mice accumulated much more CFZ in both skeletal and cardiac muscle compared with young mice. We have previously shown that CFZ progressively bioaccumulates in the lysosomes of resident macrophages [23,33]. The CFZ form present in both cardiac and skeletal muscle was predominantly the protonated form. This is the form of CFZ that is present inside acidic organelles, such as lysosomes, gets trapped in macrophages, and only clears after macrophages undergo apoptosis or when treatment is discontinued [15]. Although we did not specifically measure CFZ in skeletal or cardiac muscle macrophages, the differences in age-related accumulation may stem from slower macrophage turnover and/or changes in macrophage phenotypes that occur with increased age [34,35,36]. This phenomenon could permit greater CFZ accumulation in old mice compared with young mice. Alternative, macrophages of old mice may have a greater cargo capacity than those of young mice, since there was no age-related change in the numbers of macrophage in skeletal muscle (Figure S1C). Similarly, the higher accumulation of CFZ in cardiac muscle compared with skeletal muscle could be due to fundamental differences in the function of resident macrophages in these tissues, as described in the previous paragraph [37,38].

While our findings were unexpected and are provocative, we acknowledge there are limitations. We recognize that the measurement of l-carnitine and acetylcarnitine does not represent a comprehensive assessment of mitochondrial metabolic function. However, these metabolites are abundant and readily detectable in the blood. They are also upstream of numerous metabolic processes in the mitochondria [29,39]. As such, disruption in carnitine homeostasis was hypothesized as a reasonable gauge of mitochondrial metabolic perturbations. The duration of treatment and/or the ages studied may not have generated a significant alteration in host mitochondrial metabolic function for detection in whole blood. In previous research, CFZ altered carnitine levels in whole blood, but this study used a smaller sample and a different analytical technique (nuclear magnetic resonance spectroscopy) to quantify l-carnitine [22]. While we did not find differences in l-carnitine levels in whole blood with CFZ treatment that would have sufficiently altered the ratio of mitochondrial-related metabolites, we were able to detect an age- and tissue-dependent alteration in mitochondrial metabolism accompanied by CFZ-induced catabolism. However, the specific mechanism(s) that drive these differences require more detailed, physiological studies. Finally, understanding the extent to which these findings could bear relevance in humans will require clinical studies.

In conclusion, age is associated with an increased risk of ADRs and disturbed energy metabolism, which results in altered organ function; this is most often evident in tissues that require and/or use large amounts of energy [40]. While it has been proposed that the blood levels of two abundant mitochondrial metabolites could serve as surrogate biomarkers for assessing changes in mitochondrial metabolism and may potentially identify occult ADRs, we did not find measurable changes in either l-carnitine or acetylcarnitine blood levels following the administration of a known mitochondriotropic and lysosomotropic drug [5]. Nevertheless, there were clear changes in total body weight, food consumption, and exercise endurance that could be useful as functional biomarkers, which in humans could translate to the routine assessment of body weight, estimations of calorie consumption, and exercise tolerance/endurance, for stratifying patients at increased risk for ADRs. Future studies will be aimed at deciphering the organ-specific mechanisms that contribute to weight loss and decreased endurance. These future directions could also consist of cellular-based studies of carnitine shuttle enzymes and skeletal muscle composition, which could aid in pinpointing specific mechanisms. Importantly, in addition to CFZ, there are other drugs (e.g., doxorubicin, carbamazepine, haloperidol, chlorpromazine, and amiodarone) that are known mitochondrial toxicants and that accumulate in the lysosome, illustrating the value of detecting individuals at increased ADR risk [5,41]. Doxorubicin has been shown to elicit age-related ADRs such as cardiotoxicity and muscle atrophy [42,43,44]. Thus, establishing clinically actionable biomarkers of mitochondrial toxicity that could identify patients at the greatest ADR risk would represent an advancement in patient care, particularly in the elderly, who are most vulnerable to ADRs.

## Data Availability

All data described in this manuscript is available through the University of Michigan’s Deep Blue Data repository and can be found here: https://doi.org/10.7302/w73z-3b03 (accessed on 20 April 2023).

## Supplementary Materials

The following supporting information can be downloaded at: https://www.mdpi.com/article/10.3390/metabo13050671/s1, Methods: carnitine and CFZ LC-MS quantification, and preparation and analysis of skeletal muscle macrophage population. Figures: Figure S1. Macrophage population in skeletal muscle, and Figure S2. Difference between spectral muscle Raman references of CFZ- and vehicle-treated mice.
