# Supplementary material for: Clofazimine-Mediated, Age-Related Changes in Skeletal Muscle Mitochondrial Metabolites
Source: Metabolites. 2023 May 19;13(5):671. doi: 10.3390/metabo13050671 (PMC10220805; doi:10.3390/metabo13050671)
Supplement: Supplementary file 1 [file metabolites-13-00671-s001.zip › metabolites-2398891-supplementary.pdf]

Supplementary

1. Methods

1.1 Sample Preparation and L-Carnitine and Acetylcarnitine LC-MS/MS Analysis

Blood samples were diluted 1:50 (10 μL of WB in 490 μL water). Five μL of internal standard solution (l-carnitine-13C, D3 and acetylcarnitine-D3, 5 μg/mL in acetonitrile) were added into the diluted samples and mixed for 30 min at room temperature. For protein precipitation, 150 μL of acetonitrile was added to 50 μL of the sample. The mixture was vortexed for 10 min and centrifuged (3500 rpm for 10 min); the supernatant was transferred to autosampler vials for LC–MS/MS analysis.

Muscle samples were homogenized (Precellys tissue homogenizer, Bertin Technologies, Montigny-le-Bretonneux, France) with 80% N-Dimethylformamide (DMF)-PBS solution using a ratio of 5:1 volume (mL) to weight of muscle (g). Then, the muscle homogenate was diluted 50 times with water and treated using the same procedure as that for the blood samples to extract the compounds for LC–MS/MS analysis.

A Shimadzu LC-20AD HPLC system (Kyoto, Japan), and chromatographic separation of l-carnitine and acetylcarnitine was achieved using an Agilent Poroshell 120 EC-C18 column (3.0 x 100 mm, 2.7 μm at 25 °C). Five μL of the supernatant were injected per sample. The flow rate of gradient elution was 0.35 ml/min with mobile phase A (10 mM ammonium formate and 0.1% formic acid in purified deionized water) and mobile phase B (0.1% formic acid in acetonitrile). A Sciex QTOF X500R mass spectrometer equipped with an electrospray ionization source (ABI-Sciex, Toronto, Canada) in the positive-ion high resolution multiple reaction monitoring (HRMRM) mode was used for detection. Protonated molecular ions and the respective ion products were monitored at the transitions of m/z 162.11 > 103.0364 for L-Carnitine, 204.12 > 85.0250 for Acetyl-L-Carnitine, 166.14 > 103.0380 for L-Carnitine-13C, D3 and 207.14 > 85.0255 for Acetyl- L-carnitine-D3. We adjusted the instrument settings to maximize analytical sensitivity and specificity of detection, and quality control samples were run before, in the middle, and after the samples to evaluate the accuracy and intra-batch precision of the developed method.

1.2 Sample Preparation and CFZ LC-MS/MS Analysis

To measure CFZ in blood, 160 μL of verapamil (30 ng/mL) in acetonitrile (internal standard solution) and 20 μL of acetonitrile were added to 20 μL of heparinized whole blood. The mixture was vortexed for 10 min and centrifuged (3500 rpm for 10 min). Then, the supernatant was transferred to autosampler vials for LC–MS/MS analysis.

Muscle samples were homogenized (Precellys tissue homogenizer, Bertin Technologies, Montigny-le-Bretonneux, France) with 80% N-Dimethylformamide (DMF)-PBS solution using a ratio of 5:1 volume (mL) to weight of muscle (g). For protein precipitation, 160 μL of internal standard solution and 20 μL of acetonitrile were added into 20 μL of homogenized tissue. The mixture was vortexed for 10 min and centrifuged (3500 rpm for 10 min). The supernatant was transferred to autosampler vials for LC–MS/MS analysis.

A Shimadzu LC-20AD HPLC system (Kyoto, Japan) was used to measure CFZ concentration. Chromatographic separation of the tested compound was achieved using a Waters XBridage reverse phase C18 column (5 cm × 2.1 mm I.D., packed with 3.5 μm) at 25 °C. Five μL of the supernatant was injected per sample. The flow rate of gradient elution was 0.4 ml/min with mobile phase A (0.1% formic acid in purified deionized water) and mobile phase B (0.1% formic acid in acetonitrile). An AB Sciex QTrap 4500 mass spectrometer equipped with an electrospray ionization source (ABI-Sciex, Toronto, Canada) in the positive-

ion multiple reaction monitoring (MRM) mode was used for detection. Protonated molecular ions and the respective ion products were monitored at the transitions of m/z 473.0 > 431.1 for clofazimine and 455.2 > 303.2 for the internal standard. We adjusted the instrument settings to maximize analytical sensitivity and specificity of detection, and quality control samples were run before, in the middle, and after the samples to evaluate the accuracy and intra-batch precision of the developed method.

1.3 Preparation and Analysis of Skeletal Muscle Macrophage Population

Gastrocnemius (GAS) muscle specimens were isolated, washed with 1X PBS, blotted dry, embedded in Tissue-Plus Optimal Cutting Temperature (OCT) compound (4585, ThermoFisher, Waltham, MA, USA), and frozen on dry ice. To obtain transverse sections, the OCT blocks were thawed (room temperature), fixed in 10% neutral buffered formalin (24 h), and cryoprotected in 20% sucrose (24 h). Sections were then rinsed in water and embedded transversely in a (24x24x5mm) cryomold (Fisher Healthcare, Waltham, MA, USA) using a glycol-resin media (OCT, 4585, ThermoFisher). The cryoblocks were then immediately frozen in the vapor phase of a liquid nitrogen bath suspended in a metal bowl within an outer bath of chilled isopentane. Sections were obtained using a cryostat (10 m, CM3050 S, Leica BioSystems, Nussloch GmbH 2022).

Sections were fixed in 10% neutral buffered formalin (10 min) and stained using a rat monoclonal antibody (anti-F480, clone Cl:A3-1, MCA497RT, Bio-Rad, Hercules, CA, USA) (dilution of 1:400, 60 min, room temperature) on an automated immunohistochemical stainer (Biocare Intellipath, Biocare Medical, Pacheco, CA, USA) with prior blocking steps for endogenous peroxidases and non-specific binding. Negative control slides utilizing a ready-to-use normal rat serum (Rat Negative Control Sera, Innovex Biosciences, Richmond, CA, USA) in place of the primary antibody and positive control slides using mouse lymph node were concurrently assessed. Detection was performed with a commercial polymer-based, biotin-free reagent (Biocare Rat Probe and Rat-on-Mouse HRP, Biocare Medical). Hematoxylin was used as a nuclear counterstain. Cover slips were applied to slides using an aqueous mounting media.

Immunostained slides were digitized on a Leica Aperio AT2 digital slide scanner (Leica Biosystems) at resolution up to 0.5 µm/pixel at 20x magnification. Digital slide files were analyzed for F480 stained cells using the positive cell detection algorithm within the open-source program QuPath (0.4.3; University of Edinburgh, Edinburgh, Scotland, UK). Slides were positively annotated to select muscle tissues only for analysis and visible artifacts or confounding areas (folds, debris, bubble, nerve, adjacent non-muscle connective tissue) were excluded by negative annotation. RGB values for DAB and hematoxylin staining were set directly from singly-stained areas in the slides and the algorithm was optimized to detect F480 stained cells. Digital image overlays were visually checked for accuracy by a board-certified veterinary pathologist. The number of F480-stained cells was reported as number of positive cells/mm^2 tissue area. The mean (S.D.) number of F480 positive macrophages was calculated for each group of mice.

2. Results

2.1 The Number of Macrophages in Skeletal Muscle Remained Unchanged by Age or CFZ Treatment

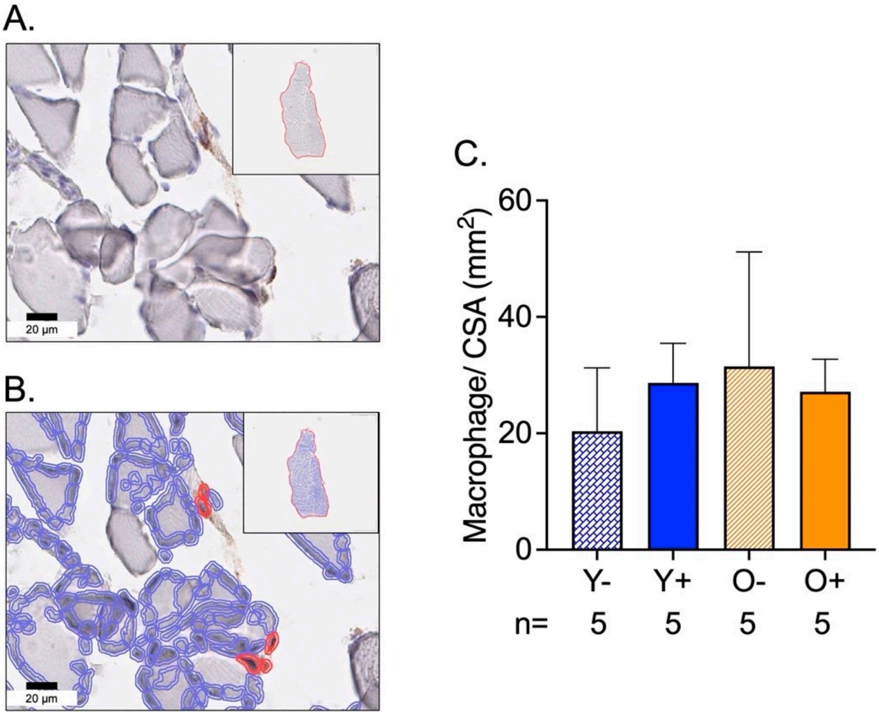

Figure S1. Macrophage population in skeletal muscle **A**. F480 positive macrophages (brown areas) and nuclei (dark circular spots) in a representative immunostained gastrocnemius (GAS) muscle section (purple areas).

B. F480 and nuclei immunostained annotated GAS section with false-color digital overlay. Red outline = macrophage positive cells, purple outline = macrophage negative cells. Both **A.** and **B.** contain total tissue overview insets for the same section. **C**. Number of F480 positive macrophages/mm^2 (mean ± SD) was not different among the groups ($p=0.53$, one-way ANOVA, n=5/group). The number of mice per group is shown below the x-axis of the plot. CSA= cross-sectional area

2.2 Spectral Differences Reveal no CFZ-FB in Muscle

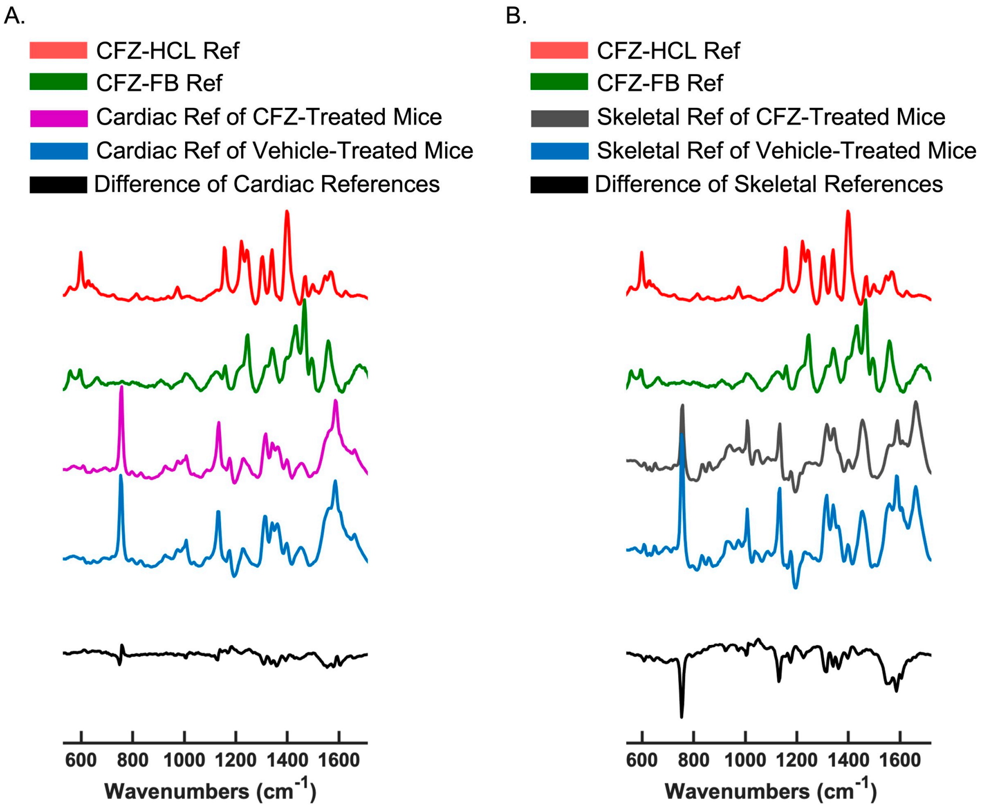

Figure S2. Difference Between Spectral Muscle Raman References of CFZ- and Vehicle-Treated Mice A. Cardiac muscle spectra (CFZ-treated mice CFZ-HCL (-) areas= purple, vehicle-treated mice =blue, difference between CFZ and vehicle treated mice sections= black). The difference spectrum shows no peaks from CFZ-HCL (red) or CFZ-FB (green) B. Skeletal muscle spectra CFZ-treated mice CFZ-HCL (-) areas= grey, vehicle-treated mice =blue, difference between CFZ and vehicle treated mice sections= black). The difference spectra show no peaks of CFZ-HCL (red) or CFZ-FB (green).
